# Case Report: A case of toothache of cardiac origin with a long-term clinical course

**DOI:** 10.3389/fpain.2025.1625582

**Published:** 2025-10-14

**Authors:** Chizuko Maeda, Takayuki Suga, Kiyotoshi Oishi, Akira Toyofuku

**Affiliations:** ^1^ Department of Psychosomatic Dentistry, Graduate School of Medical and Dental Sciences, Institute of Science Tokyo, Tokyo, Japan; ^2^ Department of Cardiovascular Surgery, Graduate School of Medical and Dental Sciences, Institute of Science Tokyo, Tokyo, Japan

**Keywords:** atypical odontalgia, angina pectoris, referred craniofacial pain, toothache differential diagnosis, toothache of cardiac origin

## Abstract

**Background:**

Toothache of cardiac origin is a rare but significant form of referred pain originating from cardiac pathology such as angina pectoris. Although jaw and throat discomfort are known referred pain sites, toothache alone is an uncommon presentation. Misdiagnosis often leads to unnecessary dental interventions and delays in appropriate cardiac treatment, highlighting the need for greater awareness among both dentists and internists.

**Case presentation:**

A 76-year-old woman presented with persistent pain in the gingiva around teeth #33 and #34, accompanied by sharp chest discomfort which would subside in about 5–6 min. Extensive dental examinations, including extractions, failed to resolve her symptoms. Initial cardiac evaluations—electrocardiogram, Holter monitoring, echocardiography, and chest computed tomography—were unremarkable. Consequently, she was diagnosed with atypical odontalgia and prescribed antidepressants, but these proved ineffective. However, over several months, the toothache worsened upon exertion, accompanied by chest pain unresponsive to standard analgesics. A specialized cardiac imaging center finally detected severe stenosis (90%–99%) of the left anterior descending artery and Right Coronary Artery, as well as a left ventricular thrombus. Coronary angiography confirmed unstable angina, and the patient underwent a Dor procedure to remove the thrombus alongside coronary artery bypass grafting. Following surgery, her toothache and chest pain completely resolved.

**Conclusion:**

This case features a protracted course from symptom onset to definitive treatment. In older patients reporting persistent tooth or gingival pain with intermittent chest discomfort—especially when symptoms are exertional and dental findings are negative—clinicians should consider a cardiac origin and expedite cardiologic imaging to avert hazardous delays. Systematic accumulation of cases and cross-disciplinary research are essential to establish actionable diagnostic guidance and move beyond anecdotal evidence.

## 1 Background

Toothache of cardiac origin refers to referred pain that presents in the teeth, oral cavity, or other facial structures, yet originates not from a dental disease but from cardiac pathology such as angina pectoris (1). In other words, although the patient complains of tooth pain, there is actually no abnormality in the teeth or gingiva, and the pain caused by myocardial ischemia is projected onto the dental region (2). To avoid ambiguity, we consistently use “toothache of cardiac origin” to denote dental pain referred from myocardial ischemia (also termed “cardiogenic toothache” in some reports). Although such tooth pain originating from the heart is rare, it does indeed exist, and when a patient experiences tooth pain despite the absence of any identifiable dental cause, the possibility of cardiac disease should be taken into account.

In cases of angina pectoris and myocardial infarction, it is well known that a typical chest pain arises in the center to the left side of the chest, radiating to the left arm, shoulder, neck, and jaw (2). Although it is relatively uncommon for this referred pain to manifest as toothache, some case reports do exist (3). In fact, it has been reported that approximately 40% of patients with myocardial ischemia experience some form of oral and facial pain (such as jaw or throat pain), and about 4% of myocardial infarction patients present with oral and facial pain as their only symptom (4). Such atypical presentations that lack chest pain tend to be more common in women, contributing to delays in diagnosis (5). Reports indicate that 8%–30% of myocardial infarctions lack chest pain, meaning that toothache alone, though rare, is not a symptom to be overlooked (5).

While instances of angina pectoris presenting solely as toothache are few—mainly appearing sporadically in the form of case reports—many have shown delays in diagnosis and mismanagement (e.g., unnecessary dental procedures) (3, 5). Because toothache caused by angina pectoris is a highly atypical symptom, it is difficult to diagnose and easily leads to inappropriate initial management (2). Indeed, in reported cases of tooth/facial pain originating in the heart, patients often undergo repeated dental treatment before a correct diagnosis is made, and delays in treatment have sometimes resulted in acute myocardial infarction (2). A unique feature of the case we experienced is that there was a long interval between the onset of toothache and chest pain before the final diagnosis of angina. Furthermore, at the patient's primary care internal medicine clinic, angina was never raised as a possible cause of her toothache, and as a result, a cardiac origin appears to have gone unnoticed. This case is presented here in hopes of preventing similar situations from recurring, serving as a reminder of the need for caution. The participant provided written informed consent, including consent for publication, prior to enrollment. The study protocol was reviewed and approved by the Tokyo Medical and Dental University Hospital Ethical Committee (approval number: D2013-005-04), and the study was conducted in accordance with the principles set forth in the Declaration of Helsinki.

## 2 Case report

A 76-year-old woman presented to Tokyo Medical and Dental University Hospital in January 2023 upon referral by both an internist and a dentist. Her chief complaint was that when she felt pain in the gingiva around #33 and #34, she experienced a sharp, stabbing pain in her chest and back, which would subside in about 5–6 min. She reported onset of similar symptoms around #34 in June 2022. In August 2022, she was brought to the emergency department for the same symptoms, but no abnormalities were detected in internal medicine examinations. Subsequently, #34 and #42 were extracted in September 2022, yet the pain persisted around #34. In December 2022, she received a denture, but the symptoms worsened, leading to her referral to our department.

**Figure 1:**
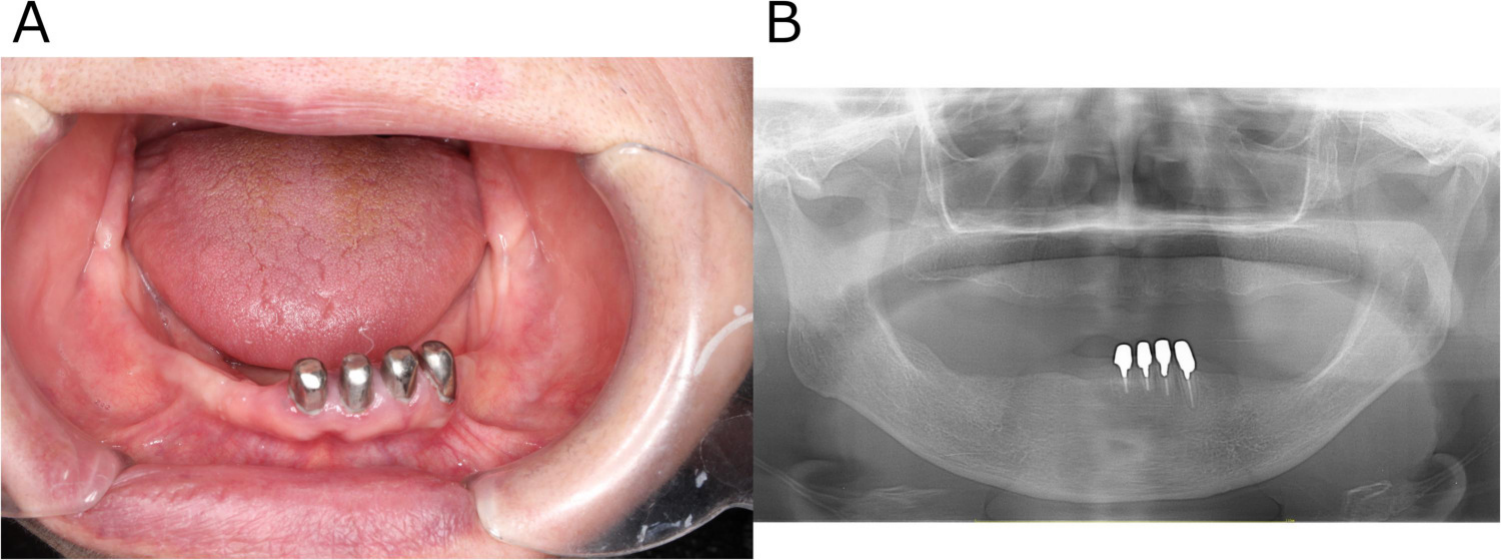
Clinical images obtained at the patient's initial visit to the psychosomatic dentistry clinic. **(A)** Intraoral photograph showing no structural or organic abnormalities that could explain the reported pain. **(B)** Panoramic radiograph likewise revealing no structural or organic abnormalities responsible for the pain.

Her past medical history included lung cancer, meningioma, and hypertension. Regarding family/social history, her daughter had a history of depression; the patient herself ran a small business selling lactic acid probiotic beverages and lived with her husband and grandchild. Oral examination revealed no signs of infection such as swelling or redness of the gingiva around #33 and #34 (Figure 1A). Sometimes the pain subsided with loxoprofen. According to the short-form McGill pain questionnaire, she selected “Throbbing”, “Sharp”, “Cramping”, “Heavy”, and “Tiring-exhausting” as “moderate”, while she answered “burning” as “none” (6). The Somatic Symptom Scale-8 (SSS-8) score was 14, indicating a high burden of somatic symptoms (7). A panoramic radiograph revealed no organic abnormalities that could be the cause of her condition (Figure 1B). The referring internist noted that 12-lead ECG, Holter ECG, echocardiography, and chest CT showed no obvious abnormalities to explain her chest pain. Table 1 shows the blood test results from August 2022. Routine laboratory tests showed no abnormalities at first presentation. If this had been the acute phase of myocardial infarction, cardiac enzymes might have been elevated, but they were not in this case. In our department, she was diagnosed with “atypical odontalgia” affecting #33 and #34.

**Table 1:** Hematological laboratory data.

Test item	Measured value	Reference range	Units
C-reactive protein (CRP)	0.01	≤0.3	mg/dl
Total protein	7.1	6.5–8.2	g/dl
Total bilirubin	1.0	0.3–1.2	mg/dl
Direct bilirubin	0.3	≤0.4	mg/dl
Aspartate aminotransferase (AST/GOT)	38	10–40	U/L
Alanine aminotransferase (ALT/GPT)	53 (H)	5–45	U/L
Alkaline phosphatase (ALP, IFCC)	108	38–113	U/L
Lactate dehydrogenase (LDH, IFCC)	160	124–222	U/L
Gamma-glutamyl transferase (*γ*-GT)	38	≤48	U/L
Creatine kinase (CK)	57	50–210	U/L
Serum amylase (AMY)	44	39–134	U/L
Sodium (Na)	144	135–145	mEq/L
Potassium (K)	3.8	3.5–5.0	mEq/L
Chloride (Cl)	110 (H)	98–108	mEq/L
Blood urea nitrogen (BUN)	14.9	8.0–20.0	mg/dl
Creatinine	0.51	0.46–0.82	mg/dl
Estimated GFR (eGFR)	87		ml/min/1.73 m^2^
Uric acid	4.5	2.7–7.0	mg/dl
Albumin	4.1	3.8–5.2	g/dl
Blood glucose	101	70–109	mg/dl
Troponin T	0.012	≤ 0.016	ng/ml
White blood cell count (WBC)	6,800	3,500–9,700	/µl
Red blood cell count (RBC)	515	376–516	×10^4^/µl
Hemoglobin (Hb)	14.9	11.2–15.2	g/dl
Hematocrit (Ht)	45.4 (H)	34.3–45.2	%
Platelet count (Plt)	34.5	14–37.9	×10^4^/µl
Mean corpuscular volume (MCV)	88.3	80–101	fl
Mean corpuscular hemoglobin (MCH)	28.9	26.4–34.3	pg
Mean corpuscular hemoglobin concentration (MCHC)	32.7	31.3–36.1	g/dl
WBC differential	–	–	–
Neutrophils (NEUTRO)	56.90%	–	%
Eosinophils (EOSINO)	4.70%	–	%
Basophils (BASO)	0.60%	–	%
Lymphocytes (LYMPH)	30.60%	–	%
Monocytes (MONO)	7.20%	–	%
N-terminal pro B-type natriuretic peptide	463 (H)	≤125	pg/ml

**Figure 2:**
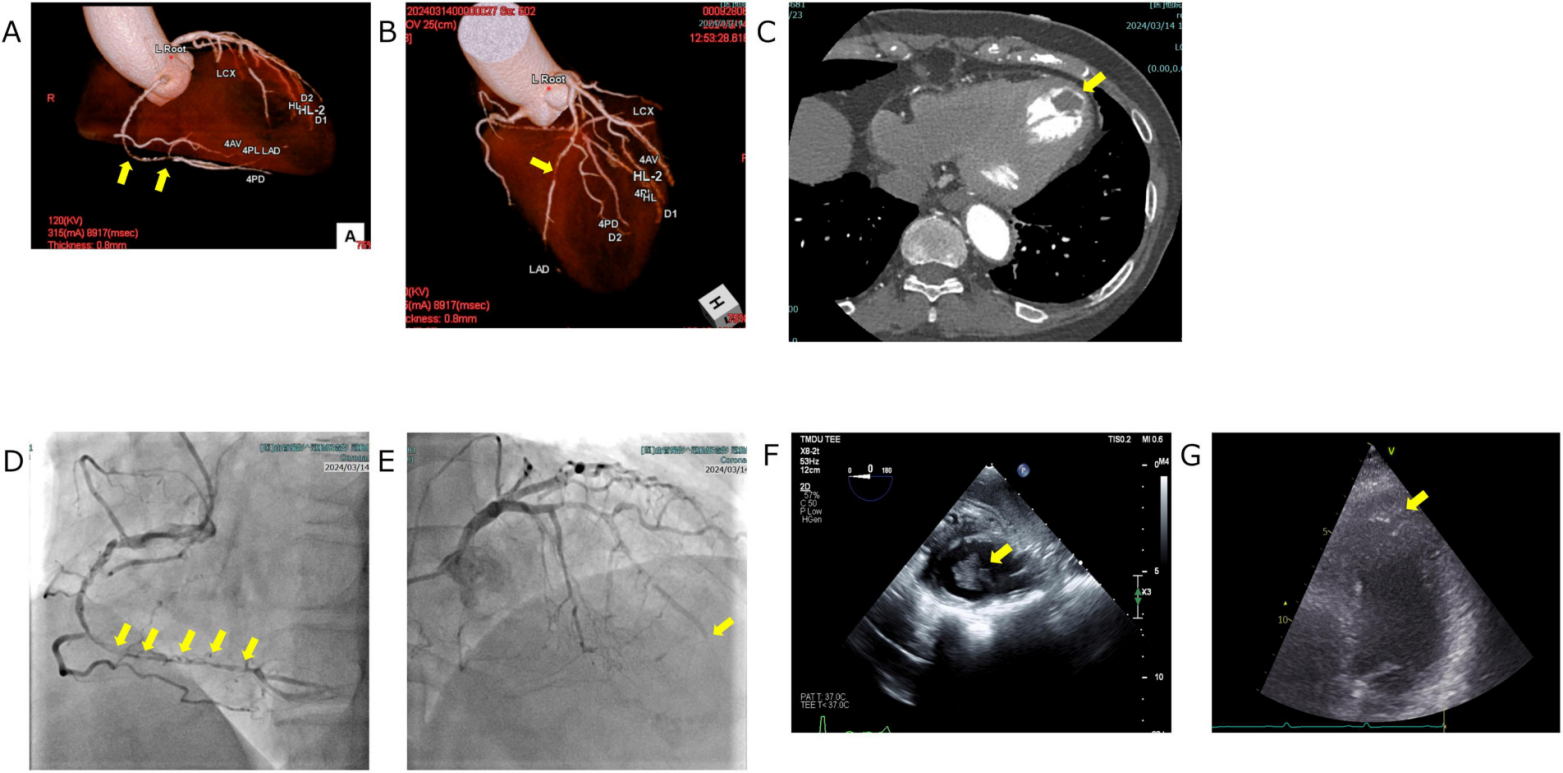
Cardiovascular imaging. **(A,B)** Coronary CT angiography depicting focal coronary stenoses (yellow arrows). **(A)** Volume-rendered view of the right coronary artery demonstrates a discrete mid-segment narrowing. **(B)** Three-dimensional reconstruction of the left coronary system shows a critical lesion in the proximal left anterior descending artery. **(C)** Axial contrast-enhanced chest CT illustrating a left-ventricular thrombus (yellow arrow). The thrombus appears as a well-defined hypodense filling defect adherent to the endocardial surface of the left ventricle. **(D, E)** Coronary angiography depicting critical coronary lesions (yellow arrows). **(D)** Right coronary artery injection demonstrates a series of tight, tandem stenoses extending from the mid- to distal vessel. **(E)** Left coronary angiogram shows an abrupt cut off of the left anterior descending artery arising from the left coronary artery, indicating complete occlusion at the site marked by the yellow arrow. **(F,G)** Echocardiographic visualization of a left ventricular thrombus: on both transesophageal **(F)** and transthoracic **(G)** views, the thrombus appears as a well-defined echogenic mass attached to the thinned, akinetic/dyskinetic endocardial surface and acoustically distinct from the blood pool (yellow arrows).

In our department, various antidepressants were prescribed according to standard protocols, but these were not sufficiently effective. In June 2023, the patient began to complain of exertional toothache and chest pain. She reported that the symptoms were consistently provoked by walking or climbing and typically subsided within 5–6 min of rest, suggesting an exertional pattern. A chest CT performed in July 2023 revealed no abnormalities. In January 2024, her exertional toothache worsened, and she reported no relief with loxoprofen. A Holter ECG was repeated at her primary care clinic in February 2024, again showing no abnormalities. However, an evaluation at a specialized cardiac imaging center revealed 90%–99% stenosis of the left anterior descending artery (LAD) and right coronary artery on coronary CT and echocardiography, as well as a thrombus in the left ventricular apex. However, evaluation at a specialized cardiac imaging center revealed occlusion of LAD #7 and 90%–99% stenosis of right coronary artery on coronary CT, and a thrombus in the left ventricular apex on echocardiography (visualized as a well-defined echogenic mass adherent to the thinned, akinetic/dyskinetic apical endocardium and distinct from the blood pool). Figures 2A–C Coronary angiography was then performed at our hospital, leading to a diagnosis of unstable angina, and she was urgently admitted to the cardiothoracic surgery ward. Subsequent coronary angiography and echocardiography performed at our hospital showed similar findings, and the diagnosis was made that emergency surgery was indicated. Figures 2D–G A Dor procedure—left ventricular reconstruction in which the infarcted, thinned, akinetic/dyskinetic aneurysmal segment is excised and the ventricular cavity is reshaped to restore more physiologic geometry and reduce stasis-related thrombus risk—was performed to remove the apical thrombus, and coronary artery bypass grafting was carried out. Specifically, a bypass from the Left Internal Thoracic Artery to LAD was created, and a bypass from the Saphenous Vein to the Posterior Descending artery was formed. Preoperative D-Dimer was 1.1 μg/ml, creatine kinase–MB (CK-MB) 2.3 ng/ml, and troponin I 17 ng/ml; postoperatively, CK-MB was 5.3 ng/ml and troponin I 2.285 ng/ml.

After discharge, both toothache and chest pain disappeared. Even now, more than one year after surgery and two years and two months since her first visit to our department, she remains free of recurrence. A concise chronology of symptoms, investigations, management, and outcomes is provided in Table 2.

**Table 2:** Clinical timeline (June 2022–2025).

Date (YYYY-MM)	Event	Key details
2022-06	Symptom onset	Gingival pain around #34 with sharp chest/back pain lasting ∼5–6 min.
2022-08	ED visit	Internal medicine workup unremarkable.
2022-09	Dental extraction	#34 and #42 extracted; pain around #34 persisted.
2022-12	Denture	Symptoms worsened; referral initiated.
2023-01	First visit to our dept.	Exam: no intraoral infection; panoramic radiograph unremarkable; prior ECG/Holter/echo/CT “no obvious abnormalities”. Dx: atypical odontalgia; antidepressants started.
2023-06	Exertional worsening	Exertional toothache and chest pain appeared.
2023-07	Chest CT	No abnormalities.
2024-01	Symptom progression	Exertional toothache worsened; loxoprofen ineffective.
2024-02	Holter (repeat)	No abnormalities at primary clinic.
2024-03	Advanced cardiac imaging	Severe LAD/RCA stenosis (90%–99%) and LV apical thrombus detected.
2024-03	Coronary angiography & admission	Dx: unstable angina; urgent admission to cardiothoracic surgery.
2024-03	Surgery	Dor procedure (LV thrombus removal) + CABG (LITA→LAD; SVG→PDA).
2025-03	Outcome & follow-up	Toothache and chest pain resolved; >1-year recurrence-free.

## 3 Discussion

It is rare for toothache of cardiac origin to persist as long as in the present case. Previous studies have reported that the duration of symptoms ranges from 3 days to 12 months (3, 8). There is also a report of a 13-year-old girl who experienced severe toothache a few times a year starting at age 10, in which cardiac disease was not suspected due to her young age, and she ultimately died. However, in that pediatric case, the pain did not persist continuously over a long period, unlike our case (9).

The presumed mechanism of cardiogenic tooth pain involves a convergence of cardiac nociceptive signals with trigeminal pathways at the level of the spinal cord and brainstem, resulting in referred pain (10). Afferent fibers of the sympathetic and vagus nerves from the heart merge with trigeminal nociceptive pathways at the level of spinothalamic tract neurons around the upper cervical spinal cord (C2), causing the brain to misinterpret the site of pain as the teeth or jaw (11). Particularly, it has been reported that if the area of myocardial ischemia is the inferior wall, there is a higher likelihood of referred pain manifesting in the facial or dental regions, a phenomenon thought to be associated with vagal innervation of the inferior wall (5). Simply put, heart pain is expressed as tooth pain due to the overlapping neural pathways.

In this case, laboratory testing showed a mildly elevated N-terminal pro-brain natriuretic peptide (NT-proBNP) of 463 pg/ml with preserved renal function (eGFR 87 ml/min/1.73 m²). In a 75-year-old, this level suggests modest ventricular wall stress but remains below commonly used age-adjusted rule-in thresholds for heart failure. Other deviations—ALT 53 U/L, chloride 110 mEq/L, and hematocrit 45.4%—were borderline and clinically nonspecific, and myocardial necrosis biomarkers were not elevated (CK 57 U/L; troponin T 0.012 ng/ml). Accordingly, the laboratory data alone did not support ischemic heart disease; the cardiac origin of the pain became evident only when the exertional worsening pattern and subsequent coronary imaging were considered together (12).

In this case, although both tooth pain and chest symptoms appeared during roughly the same period, preliminary examinations did not detect any clear findings suggesting coronary artery disease, leading the internal medicine department to conclude there was “no abnormality”. This likely caused the significant delay in diagnosis. It is well recognized, especially in older adults, that angina or ischemic heart disease may lack typical chest pain; thus, both dentists and internists must include it in the differential diagnosis.

The frequency of oral and facial pain during myocardial ischemia is reported to be about 38% (4). Further research has suggested that distinguishing among different qualities of pain could be useful in differentiating between dental and cardiac origins; patients with cardiac oral and facial pain often report “pressure” or “burning”, while dental pain frequently involves “throbbing” or “aching” (13). However, it is crucial to recognize that these lexical pain descriptors show substantial overlap across etiologies and lack specificity; therefore, they should not be used in isolation for differential diagnosis, as they may mislead clinicians (13). In our case, the pain was often described as “Throbbing”, which favored a dental origin, and the lack of descriptors such as “burning” added to the confusion. Meanwhile, the high SSS-8 score and various complaints (skin-related, cough, etc.) hinted at psychosomatic factors, leading to the strong suspicion of “atypical odontalgia” in the absence of dental causes. These considerations likely contributed to the delay in diagnosing cardiogenic tooth pain. This case therefore illustrates how reliance on pain quality alone can be misleading. Instead, an exertional pattern—provocation by activity and relief within minutes of rest—intermittent chest/back discomfort, and non-response to dental procedures and analgesics should raise suspicion for a cardiac source and prompt early cardiology referral (2, 4, 5, 12). In retrospect, our patient's progressive exertional toothache that remained refractory to extractions, antidepressants, and analgesics represented the critical red flags for coronary ischemia. Accordingly, pain quality should be interpreted only in conjunction with other clinical information. In high-risk patients, no single symptom or descriptor—whether “throbbing”, “burning”, or otherwise—should be used to definitively exclude a cardiac source (5, 13).

### 3.1 Balancing psychosomatic burden with vigilance for organic disease

1) Balancing psychosomatic burden with vigilance for organic disease: While our patient had a high SSS-8 score and a working diagnosis of atypical odontalgia, psychosomatic burden should be treated as a modifier of risk rather than a diagnostic endpoint. The SSS-8 quantifies symptom burden (with established severity thresholds) but does not localize etiology; thus clinicians should integrate it with objective cues and predefined re-evaluation triggers. In high-risk profiles, a reproducible exertional pattern, non-response to dental therapies, or discordant biomarkers/imaging should prompt escalation to cardiac testing (e.g., stress imaging or Coronary CT Angiography), consistent with contemporary chest-pain pathways. This approach mitigates diagnostic overshadowing—misattributing organic symptoms to an existing psychosomatic label—and helps shorten time to definitive diagnosis (7, 14, 15).
2) Chemical mediators and descending modulatory pathways relevant to this case: Myocardial ischaemia release adenosine, bradykinin, protons/lactate, prostaglandins, substance P and others that activate cardiac afferents; convergence of cardiac and craniofacial inputs within brainstem nuclei provides a substrate for referred oral and facial pain. In atypical odontalgia, central sensitization and reduced descending inhibition (serotonergic/noradrenergic–opioidergic circuits from the Periaqueductal Gray–Rostroventral Medulla/Locus Coeruleus) are implicated, which can amplify pain perception and complicate interpretation of descriptors. Recognizing these parallel mechanisms explains how psychosomatic features and cardiogenic referral can co-exist, reinforcing the need for integrative assessment (16–19).

A practical approach is to integrate subjective descriptors with: (1) exertional pattern—provocation by activity and relief with rest; (2) concomitant features such as chest, back, or dyspnea symptoms; (3) lack of response to dental procedures and analgesics; and (4) initial cardiac testing when appropriate (ECG, troponins in acute settings), followed by timely cardiology referral for noninvasive imaging and, if uncertainty persists or risk is high, invasive coronary angiography in accordance with contemporary guidance (2, 4, 12, 13).

Nonetheless, certain signals could have pointed to a cardiac origin, such as the fact that the pain worsened during exertion and sometimes subsided with loxoprofen, as well as the concurrent sharp, stabbing pain in the chest. According to the 2024 ESC guidelines for chronic coronary syndromes, invasive coronary angiography via catheterization is recommended as the final diagnostic step for high-risk cases or when noninvasive tests are inconclusive (12). Ultimately, high-grade stenosis of the LAD and right coronary artery was confirmed by catheter-based imaging, and all symptoms, including toothache, resolved after cardiac surgery, making it clear that the true cause of toothache was ischemic heart disease.

In dental practice, when encountering atypical toothache accompanied by systemic symptoms such as chest discomfort or dyspnea, it is important to actively consider evaluation of the circulatory system, including possible coronary artery disease—not only psychosomatic causes. In particular, for older patients and those with significant past medical histories, a more cautious approach is warranted. Going forward, an accumulation of additional cases and multicenter collaborative research are expected to facilitate the establishment of diagnostic criteria for early detection of heart-related toothache. At the initial visit, routine blood tests were unremarkable, and there were no clinical signs of heart failure. Given the patient's symptoms and medical history, it was difficult to immediately suspect a cardiac cause. Moreover, the complaint was framed as common problems such as “toothache” and “stiff shoulder”, which can make it harder to consider other etiologies, including cardiac disease. Although history-taking is crucial, it is not feasible in daily practice to suspect myocardial ischemia and systematically ask about exertional provocation in every case. When no intraoral abnormality is found and symptoms persist, clinicians should broaden the differential diagnosis to include non-dental causes.

### 3.2 Comparative context: misdiagnosis and outcomes

In recent years, several international reports have described cardiogenic oral and facial pain that was initially misdiagnosed as odontogenic disease. Choi et al. reported a 60-year-old man with five years of bilateral jaw pain provoked by ordinary activities and relieved by rest; dental and temporomandibular imaging were unremarkable, coronary CT angiography revealed obstructive coronary artery disease, and symptoms resolved after percutaneous coronary intervention (PCI) (11). Likewise, Kawaguchi and Ichinohe described a 70-year-old woman with exertional intraoral burning pain, no chest symptoms, prior periodontal/caries management, and a 99% proximal right coronary artery stenosis; same-day PCI led to complete pain resolution (8). Compared with these cases, our patient had a longer course, a different provoking pattern (gingival pain evolving into exertional toothache with concomitant chest pain), multivessel disease with a left-ventricular thrombus, and required surgical revascularization with thrombus removal (Dor procedure plus coronary artery bypass grafting (CABG). Nonetheless, all cases share the same diagnostic pitfall: isolated oral and facial pain in the absence of dental pathology delayed recognition of myocardial ischemia.

Beyond individual cases, contemporary reviews emphasize that oral and facial pain during myocardial ischemia is common (≈40%) and, in up to 4% of MI patients, may be the sole manifestation—more often described as pressure/burning than throbbing, which may help distinguish it from dental pain (5). A recent scoping review further consolidated that acute coronary syndrome (ACS) presentations without typical cardiac chest pain are consistently associated with increased mortality and morbidity, prolonged prehospital and in-hospital delays, and less frequent use of reperfusion and evidence-based medications (20). Taken together, these data contextualize our case and underscore the clinical imperative to promptly reframe “toothache” as a potential anginal equivalent, especially in older adults and in settings where initial dental investigations are unrevealing.

## 4 Conclusions

The lesson from this case is that in patients who present with tooth pain accompanied by chest discomfort or signs of angina, one must always consider toothache of cardiac origin —even over a prolonged clinical course and in the absence of clear dental findings. By performing an appropriate differential diagnosis and identifying the true source of pain, we can prevent unnecessary procedures and serious delays in treatment. In ambiguous cases, clinicians should prioritize exertional characteristics and lack of response to dental interventions over the lexical quality of the pain when considering cardiogenic toothache. In high-risk patients, clinicians should avoid using any single symptom or lexical descriptor to rule out a cardiogenic source and instead integrate pain quality with exertional characteristics, objective data, and treatment response when forming the differential. Beyond the uniqueness of this presentation, single cases cannot establish prevalence, diagnostic performance, or outcomes. We therefore call for prospective accumulation of cases and multicenter collaborative research—ideally spanning dental and cardiology settings—to quantify the frequency of toothache of cardiac origin, to define pragmatic diagnostic red flags (e.g., exertional pattern; non-response to dental procedures), and to test streamlined pathways (e.g., early coronary CT angiography and/or contrast echocardiography). Such studies should track time to correct diagnosis, avoidance of unnecessary dental procedures, and cardiovascular outcomes. A coordinated registry would help move this topic from anecdote to actionable guidance.

## Data Availability

The original contributions presented in the study are included in the article/Supplementary Material, further inquiries can be directed to the corresponding author.
